# Association of Relatives of Hemodialysis Patients with Metabolic Syndrome, Albuminuria and Framingham Risk Score

**DOI:** 10.1371/journal.pone.0096362

**Published:** 2014-05-07

**Authors:** Jiun-Chi Huang, Szu-Chia Chen, Ming-Yen Lin, Jer-Ming Chang, Shang-Jyh Hwang, Jer-Chia Tsai, Hung-Chun Chen

**Affiliations:** 1 Division of Nephrology, Department of Internal Medicine, Kaohsiung Medical University Hospital, Kaohsiung Medical University, Kaohsiung, Taiwan; 2 Department of Internal Medicine, Kaohsiung Municipal Hsiao-Kang Hospital, Kaohsiung Medical University, Kaohsiung, Taiwan; 3 Faculty of Medicine, Kaohsiung Medical University, Kaohsiung, Taiwan; 4 Faculty of Renal Care, College of Medicine, Kaohsiung Medical University, Kaohsiung, Taiwan; Tor Vergata University of Rome, Italy

## Abstract

**Background and Aim:**

Metabolic syndrome (MetS), albuminuria, and the Framingham Risk Score (FRS) are significant predictors for cardiovascular disease (CVD). However, the relationship and clinical significance of these CVD predictors in individuals with a family history of end-stage renal disease (ESRD) are unclear. We investigated the association of relatives of hemodialysis (HD) patients with MetS, albuminuria, and the FRS.

**Methods:**

One hundred and sixty-six relatives of HD patients and 374 age- and sex- matched community controls were enrolled. MetS was defined using the Adult Treatment Panel III for Asians. Albuminuria was defined as urine albumin-to-creatinine ratio ≥30 mg/g. CVD risk was evaluated by the FRS.

**Results:**

A significantly higher prevalence of MetS (19.9% *vs.* 12.5%, *P* = 0.026), albuminuria (12.7% *vs.* 5.1%, *P* = 0.002) and high FRS risk ≥10% of 10-year risk (15.7% *vs.* 8.5%, *P* = 0.013) was found in relatives of HD patients compared to their counterpart controls. In multivariate analysis, being relatives of HD patients (*vs.* controls) was an independent determinant for MetS (odds ratio [OR], 1.785; 95% confidence interval [CI], 1.045 to 3.050), albuminuria (OR, 2.891; 95% CI, 1.431 to 5.841), and high FRS risk (OR, 1.863; 95% CI, 1.015 to 3.418). Higher low-density lipoprotein cholesterol (OR, 1.034; 95% CI, 1.017 to 1.052) and betel nut chewing (OR, 13.994; 95% CI, 3.384 to 57.871) were independent determinants for having a high FRS risk in relatives of HD patients.

**Conclusions:**

Being relatives of HD patients was independently associated with MetS, albuminuria and high FRS risk, suggesting family members of ESRD patients may have higher CVD risks through the interactions of renal risk factors. Proactive surveillance of these CVD predictors and preventive strategies should be targeted to this high-risk population.

## Introduction

Cardiovascular disease (CVD) has been recognized as the world’s major healthcare burden in recent decades, accounting for 17.3 million deaths in 2012 [1]. CVD is multifactorial in origin, and the established risk factors for CVD include family history, older age, hypertension, diabetes mellitus (DM), smoking and dyslipidemia. Several markers have been identified in clinical settings as significant predictors for CVD. A number of studies have found a correlation between albuminuria with the risk and mortality of CVD [2], [3]. Metabolic syndrome (MetS), a clustering of central obesity, hypertension, fasting hyperglycemia, and dyslipidemia, has been considered as a risk factor for type 2 DM and CVD [4], [5]. The Framingham Risk Score (FRS) is a valid assessment tool used to predict the coronary heart disease in the next 10 years after the evaluation [6], [7]. Overall, albuminuria, MetS, and the FRS could function as strong predictors for CVD in the general population [3], [4], [8].

Moreover, there is rapidly growing disease burden for chronic kidney disease (CKD) and end-stage renal disease (ESRD) that is associated with high morbidity and mortality [9]. Family members of ESRD patients were reported to be at higher risks for development of CKD or ESRD [10]–[16]. In particular, CVD is the leading cause of death in this population [17]. The clinical significance and the relationship of the above-mentioned predictors for CVD in individuals with a family history of ESRD remain unclear. Current clinical guidelines stress the importance of identifying individuals at excess risk for CVD [18]. Therefore, the aim of our study was to evaluate the association of relatives of hemodialysis (HD) patients with MetS, albuminuria, and the FRS.

## Materials and Methods

### Ethics Statement

The study protocol was approved by the institutional review board of the Kaohsiung Medical University Hospital (KMUH-IRB-950026). Written informed consents were obtained from each patient, and all clinical investigation was conducted according to the principles expressed in the Declaration of Helsinki. The patients gave consent for the publication of the clinical details.

### Study Design and Participants

Our study included 190 first- and second-degree relatives of 93 index HD patients from Kaohsiung City area, Taiwan with dialysis duration of 3 months to 21 years. The underlying causes of HD were chronic glomerular disease (n = 47; 50.5%), diabetic nephropathy (n = 28; 30.1%), tubulointerstitial disease (n = 7; 7.5%), hypertension (n = 7; 7.5%) and other renal diseases (n = 4; 4.3%). HD patients with inherited kidney disease, such as autosomal dominant polycystic kidney disease, were excluded. Among the 190 relatives of HD patients, 16 were excluded from study because of refusal, loss of contact, or lack of information.

Controls were selected from a population that participated in a community screening program that consisted of 2,762 individuals in the Kaohsiung City area in 2005 (men/women, 771/1,991; mean age, 52.0±13.2 years). The number of participants in each age-stratified group (≤40, 41 to 65, and >65 years) were 766, 1,524 and 472, respectively. In selecting controls for the HD relatives, 380 sex- and age-stratified matched persons without a family history of ESRD were randomly selected as controls. For calculating the FRS, individuals aged <20 or ≥80 years were excluded for the study. Finally, there were 166 relatives of HD patients and 374 controls enrolled and analyzed.

### Collection of Demographic, Medical and Laboratory Data

Demographic and medical data including age, gender, smoking history (current or non-smoker), personal history of betel nut chewing (ever or never), personal and family history of kidney diseases, and comorbid conditions were obtained from medical records and interviews with all participants.

Anthropometric measurements were obtained using standard protocols and techniques. Waist circumference was measured using a tape measure mid-way between the lowest rib and the iliac crest with the subject standing. Venous blood was collected after an overnight fast for measurement of various biomarkers: fasting blood glucose, serum creatinine, total cholesterol, triglycerides, high-density lipoprotein cholesterol (HDL-C), low-density lipoprotein cholesterol (LDL-C), and serum uric acid. Biochemical data were measured using an autoanalyzer (COBAS Integra 400 plus; Roche Diagnostics, www.roche.com/diagnostics/). Urinary albumin and creatinine were measured from one spot urine sample using the same analyzer.

### Definition of DM and Hypertension

DM was defined as a fasting blood glucose level ≥126 mg/dL, a previous diagnosis of diabetes by a physician, or use of lowing hyperglycemic agents. Blood pressure (BP) was measured using a mercury sphygmomanometer with the participant at rest in a seated position for at least 5 minutes. Hypertension was defined as a systolic BP≥140 mmHg, diastolic BP≥90 mmHg, previous diagnosis of hypertension by a physician, or use of anti-hypertensive medication.

### Estimated Glomerular Filtration Rate (eGFR) and Albuminuria

The eGFR was measured using the four-variable equation in the Modification of Diet in Renal Disease Study [19]. eGFR ml/min/1.73 m^2^ = 186 × Serum creatinine ^−1.154^ × Age ^−0.203^ × 0.742 (if female). Albuminuria was defined as urinary albumin to creatinine ratio ≥30 mg/g.

### Definition of MetS

MetS was defined as requiring the presence of any three of the following five components based on the standard of the National Cholesterol Education Program-Adult Treatment Panel III (NCEP-ATP III) [20] and modified for Asians [21]: (1) central obesity, defined as a waist circumference >90 cm for men and >80 cm for women; (2) raised BP, defined as a systolic BP≥130 mmHg, diastolic BP≥85 mmHg, or physician-diagnosed or -treated hypertension; (3) raised triglycerides, defined as a triglycerides concentration ≥150 mg/dL; (4) low HDL-C, defined as an HDL-C concentration <40 mg/dL in men and <50 mg/dL in women; and (5) fasting hyperglycemia, defined as a fasting whole-blood glucose concentration ≥110 mg/dL or DM.

### FRS Calculation and Risk Category

The FRS was calculated from the NCEP-ATP III algorithm based on six coronary risk factors: gender, age, total cholesterol, HDL-C, systolic BP, and smoking habit [7]. Among these factors, age, BP, and cholesterol levels were categorized according to their values, and smoking status was classified as either “current smoker” or “non-smoker”. The FRS was used to identify individuals categorically as either “low” risk (<10% 10-year risk) or “high” risk (≥10% 10-year risk).

### Statistical Analysis

All statistical analyses were performed using SPSS software version 17.0 (SPSS Inc, Chicago, IL, USA). The data are expressed as mean ± SD for continuous variables, percentage for categorical variables, or median (25^th^–75^th^ percentile) for triglycerides. Differences in variables between the two groups were analyzed using the Chi-square test for categorical variables or the independent *t-*test for continuous variables. The multiple logistic regression analysis with forward selection approach was used to identify the factors associated with the risk for MetS, albuminuria and high FRS risk. The significant variables in the univariate analysis were selected for multivariate analysis. A difference was considered significant when *P*<0.05.

## Results

Table 1 summarizes the baseline clinical characteristics of relatives of HD patients and community controls. There were no differences in the prevalence of diabetes, history of betel nut chewing, waist circumference, fasting glucose, triglyceride, total cholesterol, LDL-C, uric acid, or eGFR between these two groups. Among controls, 22 (5.9%) participants had a family history of CKD and none had a family history of ESRD. Compared to community controls, relatives of HD patients had a higher prevalence of hypertension and smoking habit, higher systolic and diastolic BPs, and lower HDL-C. In addition, while the mean age of relatives of HD patients was significantly younger than that of controls (38.1±12.0 *vs*. 41.2±12.7 years; *P* = 0.012), the prevalence of MetS (19.9% *vs.* 12.5%; *P* = 0.026), albuminuria (12.7% *vs.* 5.1%; *P* = 0.002) and high FRS risk (15.7% *vs.* 8.5%; *P = *0.013) was significantly higher among the relatives.

**Table 1:** Comparison of baseline characteristics between relatives of HD patients and community controls.

Variables	Relatives of HD patients (n = 166)	Community controls (n = 374)	*P* value
Age (year)	38.1±12.0	41.2±12.7	0.012
Male gender (%)	47.0	47.7	0.873
Diabetes mellitus (%)	2.4	1.9	0.744
Hypertension (%)	28.3	19.2	0.018
Smoking (%)	23.5	11.2	<0.001
Betel nut chewing (%) (ever *vs.* never)	7.9	4.8	0.157
Waist circumference (cm)	78.7±13.6	79.5±10.6	0.538
Systolic BP (mmHg)	124.3±19.2	120.2±16.9	0.009
Diastolic BP (mmHg)	79.9±13.7	76.3±11.1	0.002
Laboratory parameters			
Glucose (mg/dL)	89.7±18.9	86.7±21.9	0.124
Triglycerides (mg/dL)	108.7 (83.0–156.4)	98.8 (74.7–154.1)	0.221
Total cholesterol (mg/dL)	202.1±35.5	197.9±37.4	0.188
HDL-C (mg/dL)	55.9±14.5	59.7±14.3	0.007
LDL-C (mg/dL)	124.7±31.6	125.1±34.2	0.749
Uric acid (mg/dL)	6.0±1.7	5.8±1.6	0.084
eGFR (ml/min/1.73 m^2^)	82.3±12.3	83.9±12.9	0.149
MetS (%)	19.9	12.5	0.026
Albuminuria (%)	12.7	5.1	0.002
High FRS risk (%)	15.7	8.5	0.013

Abbreviation: HD, hemodialysis; BP, blood pressure; HDL-C, high-density lipoprotein cholesterol; LDL-C, low-density lipoprotein cholesterol; eGFR, estimated glomerular filtration rate; MetS, metabolic syndrome; FRS, Framingham Risk Score.

The FRS is used to identify individuals categorically as “low” (<10% of 10-year risk), or “high” risk (≥10% of 10-year risk).

### Determinants of MetS in All Subjects

Table 2 shows the determinants of MetS in all subjects. In the univariate regression analysis, MetS was significantly associated with relatives of HD patients, older age, male gender, smoking habit (current *vs.* non-smoker), higher total cholesterol, LDL-C, and uric acid, lower eGFR and albuminuria. The multivariate forward analysis revealed that being relatives of HD patients (*vs.* community controls; odds ratio [OR], 1.785; 95% confidence interval [CI], 1.045 to 3.050; *P* = 0.034), older age (OR, 1.045; 95% CI, 1.024 to 1.066; *P*<0.001), having higher uric acid (OR, 1.440; 95% CI, 1.244 to 1.668; *P*<0.001), and albuminuria (OR, 2.196; 95% CI, 1.004 to 4.803; *P* = 0.049) were independently associated with MetS.

**Table 2:** Determinants of MetS in all subjects.

Parameter	Univariate		Multivariate (Forward)	
	OR (95% CI)	*P*	OR (95% CI)	*P*
Relatives of HD patients *vs.* Community controls	1.732 (1.062–2.823)	0.028	1.785 (1.045–3.050)	0.034
Age (per 1 year)	1.040 (1.021–1.060)	<0.001	1.045 (1.024–1.066)	<0.001
Male gender	1.510 (0.936–2.436)	0.091	–	–
Smoking (current *vs.* non-smoker)	2.004 (1.122–3.581)	0.019	–	–
Betel nut chewing (ever *vs.* never)	1.770 (0.736–4.260)	0.202	–	–
Total cholesterol (per 1 mg/dL)	1.010 (1.003–1.016)	0.003	–	–
LDL-C (per 1 mg/dL)	1.006 (0.999–1.013)	0.117	–	–
Uric acid (per 1 mg/dL)	1.417 (1.231–1.630)	<0.001	1.440 (1.244–1.668)	<0.001
eGFR (per 1 ml/min/1.73 m^2^)	0.952 (0.933–0.972)	<0.001	–	–
Albuminuria	3.119 (1.534–6.343)	0.002	2.196 (1.004–4.803)	0.049

Values expressed as odds ratio (OR) and 95% confidence interval (CI).

Abbreviations are the same as in Table 1.

### Determinants of Albuminuria in All Subjects

Table 3 shows the determinants of albuminuria in all subjects. The univariate regression analysis revealed that albuminuria was significantly associated with relatives of HD patients, older age, DM, hypertension, lower eGFR, and the MetS. The multivariate forward analysis revealed that being relatives of HD patients (*vs.* community controls; OR, 2.891; 95% CI, 1.431 to 5.841; *P* = 0.003), older age (OR, 1.029; 95% CI, 1.000 to 1.059; *P* = 0.046), male gender (*vs.* female; OR, 0.438; 95% CI, 0.209 to 0.914; *P* = 0.028), DM (OR, 7.860; 95% CI, 2.051 to 30.119; *P* = 0.003) and hypertension (OR, 3.051; 95% CI, 1.436 to 6.483; *P* = 0.004) were independently associated with albuminuria.

**Table 3:** Determinants of albuminuria in all subjects.

Parameter	Univariate		Multivariate (Forward)	
	OR (95% CI)	*P*	OR (95% CI)	*P*
Relatives of HD patients *vs.* Community controls	2.714 (1.417–5.198)	0.003	2.891 (1.431–5.841)	0.003
Age (per 1 year)	1.037 (1.012–1.063)	0.004	1.029 (1.000–1.059)	0.046
Male gender	0.572 (0.292–1.121)	0.103	0.438 (0.209–0.914)	0.028
Diabetes mellitus	11.786 (3.427–40.537)	<0.001	7.860 (2.051–30.119)	0.003
Hypertension	4.061 (2.104–7.838)	<0.001	3.051 (1.436–6.483)	0.004
Smoking (current *vs.* non-smoker)	1.002 (0.407–2.470)	0.996	–	–
Betel nut chewing (ever *vs.* never)	1.408 (0.408–4.855)	0.588	–	–
Triglycerides (per log 1 mg/dL)	2.973 (0.840–10.523)	0.091	–	–
Total cholesterol (per 1 mg/dL)	1.006 (0.097–1.014)	0.119	–	–
HDL-C (per 1 mg/dL)	0.985 (0.962–1.009)	0.221	–	–
LDL-C (per 1 mg/dL)	1.008 (0.999–1.017)	0.098	–	–
Uric acid (per 1 mg/dL)	1.048 (0.866–1.267)	0.633	–	–
eGFR (per 1 ml/min/1.73 m^2^)	0.963 (0.938–0.989)	0.005	–	–
MetS	3.119 (1.534–6.343)	0.002	–	–

Values expressed as odds ratio (OR) and 95% confidence interval (CI).

Abbreviations are the same as in Table 1.

### Determinants of High FRS Risk in All Subjects

Table 4 shows the determinants of high FRS risk in all subjects. In the univariate regression analysis, high FRS risk was significantly associated with relatives of HD patients, betel nut chewing, DM, higher triglyceride, higher LDL-C, higher uric acid, lower eGFR, MetS and albuminuria. The multivariate forward analysis revealed that being relatives of HD patients (*vs.* community controls; OR, 1.863; 95% CI, 1.015 to 3.418; *P* = 0.045), betel nut chewing (ever *vs.* never) (OR, 5.059; 95% CI, 2.073 to 12.347; *P*<0.001), higher triglyceride (OR, 8.238; 95% CI, 2.413 to 28.121; *P* = 0.001), higher LDL-C (OR, 1.016; 95% CI, 1.007 to 1.025; *P*<0.001), and lower eGFR (OR, 0.963; 95% CI, 0.940 to 0.987; *P* = 0.002) were independently associated with high FRS risk.

**Table 4:** Determinants of high FRS risk in all subjects.

Parameter	Univariate		Multivariate (Forward)	
	OR (95% CI)	*P*	OR (95% CI)	*P*
Relatives of HD patients *vs.* Community controls	1.991 (1.144–3.463)	0.015	1.863 (1.015–3.418)	0.045
Betel nut chewing (ever *vs.* never)	5.536 (2.498–12.267)	<0.001	5.059 (2.073–12.347)	<0.001
Diabetes mellitus	3.239 (0.835–12.567)	0.089	–	–
Triglycerides (per log 1 mg/dL)	13.851 (4.668–40.924)	<0.001	8.238 (2.413–28.121)	0.001
LDL-C (per 1 mg/dL)	1.016 (1.008–1.024)	<0.001	1.016 (1.007–1.025)	<0.001
Uric acid (per 1 mg/dL)	1.366 (1.169–1.596)	<0.001	–	–
eGFR (per 1 ml/min/1.73 m^2^)	0.952 (0.930–0.974)	<0.001	0.963 (0.940–0.987)	0.002
MetS	3.370 (1.830–6.206)	<0.001	–	–
Albuminuria	2.678 (1.205–5.951)	0.016	–	–

Values expressed as odds ratio (OR) and 95% confidence interval (CI).

Abbreviations are the same as in Table 1.

The FRS is used to identify individuals categorically as “low” (<10% of 10-year risk), or “high” risk (≥10% of 10-year risk).

### Determinants of MetS, Albuminuria, and High FRS Risk in Relatives of HD Patients

Table 5 shows that in the multivariate forward analysis, independent determinants for MetS in relatives of HD patients were older age (OR, 1.044; 95% CI, 1.009 to 1.079; *P* = 0.014) and higher uric acid (OR, 1.565; 95% CI, 1.220 to 2.007; *P*<0.001). For albuminuria, the independent determinants in the relatives were hypertension (OR, 3.535; 95% CI, 1.275 to 9.804; *P* = 0.015) and lower eGFR (OR, 0.947; 95% CI, 0.905 to 0.991; *P* = 0.019). For high FRS risk, the independent determinants in the relatives were higher LDL-C (OR, 1.034; 95% CI, 1.017 to 1.052; *P*<0.001) and betel nut chewing (ever *vs.* never) (OR, 13.994; 95% CI, 3.384 to 57.871; *P*<0.001).

**Table 5:** Determinants of MetS, albuminuria, and high FRS risk in relatives of HD patients.

	Multivariate (Forward)	
MetS	Adjusted OR (95% CI)	*P*
Age (per 1 year)	1.044 (1.009–1.079)	0.014
Uric acid (per 1 mg/dL)	1.565 (1.220–2.007)	<0.001
**Albuminuria**	**Adjusted OR (95% CI)**	***P***
Hypertension	3.535 (1.275–9.804)	0.015
(per 1 ml/min/1.73 m^2^)	0.947 (0.905–0.991)	0.019
**High FRS risk**	**Adjusted OR (95% CI)**	***P***
LDL-C (per 1 mg/dL)	1.034 (1.017–1.052)	<0.001
Betel nut chewing (ever *vs.* never)	13.994 (3.384–57.871)	<0.001

Values expressed as odds ratio (OR) and 95% confidence interval (CI).

For MetS: adjusted for age, sex, smoking, betel nut chewing, total cholesterol, LDL-C, uric acid, eGFR, and albuminuria.

For albuminuria: adjusted for age, sex, diabetes mellitus, hypertension, smoking, betel nut chewing, total cholesterol, HDL-C, LDL-C, log-transformed triglycerides, uric acid, eGFR, and MetS.

For high FRS risk: adjusted for betel nut chewing, diabetes mellitus, log-transformed triglycerides, LDL-C, uric acid, eGFR, albuminuria, and MetS.

Abbreviations are the same as in Table 1.

The FRS is used to identify individuals categorically as “low” (<10% of 10-year risk), or “high” risk (≥10% of 10-year risk).

## Discussion

There are several important findings in the present study. First, we found that when compared to normal controls, being relatives of HD patients was independently associated with a higher prevalence of MetS, albuminuria and higher predicting CVD based on higher FRS risk. Previous studies revealed that a family history of ESRD was associated with not only a higher prevalence of albuminuria and proteinuria [22], but also with an increased risk of developing CKD or ESRD [10]–[16]. However, few studies have addressed the relationship between MetS and cardiovascular risks in this population. A strong association between albuminuria and MetS was reported in the Third National Health and Nutrition Examination Survey (NHANES III) [23]. The risk for albuminuria was increased along with the number of traits of MetS [23]. Palaniappan et al. [24] also reported a higher risk for albuminuria in both women and men with MetS compared to those without it. One important finding of our study was that relatives of HD patients were independently associated with MetS and albuminuria. Independent determinants for MetS in relatives of HD patients include older age, hyperuricemia and albuminuria. Independent determinants for albuminuria include older age, female gender, diabetes, and hypertension. The causal relationship between MetS and albuminuria is not fully understood. In individuals with MetS, the pathologic abnormalities of the kidneys showed increased microvascular disease, tubular atrophy, interstitial fibrosis, global and segmental glomerulosclerosis [25]. Evidence indicates that the constellation of insulin resistance, hypertension, dyslipidemia, and inflammation might cause renal injury in individuals with MetS [26].

Furthermore, previous studies have shown familial clustering of MetS-related traits, such as obesity, hypertension, insulin resistance, and dyslipidemia [27], [28]. Individuals with a family history of ESRD were reported to be more likely to have hypertension, diabetes, obesity, and proteinuria [12], [29]. This implies that the genetic traits, shared environmental and behavioral factors may contribute to development of MetS in relatives of HD patients.

Our finding of increased prevalence of albuminuria (12.7%) among the relatives of ESRD patients is consistent with earlier studies, with results ranging from 9.5% to 19.2% [10], [30], [31]. However, the age of relatives in the present study was relatively young. Albuminuria is a known marker of endothelial dysfunction and vascular leakiness [32]. Accumulating evidence has revealed that endothelial dysfunction is an important component of insulin resistance and the MetS [33], which may also play a central role in the pathogenesis of atherosclerosis, leading to CVD and adverse cardiovascular outcomes [34], [35]. It suggested that screening programs and intervention should be performed as early as possible to decrease the burden of CVD in this population.

Another important finding of our study was that relatives of HD patients were independently associated with high FRS risk. The FRS allows for estimation of the individual’s 10-year risk of coronary heart disease by using traditional cardiac risk factors including age, gender, systolic BP, total cholesterol, HDL-C, and smoking status [7]. Previous studies found familial aggregation of traditional CVD risk factors, such as hypertension, diabetes, dyslipidemia, smoking and obesity among family members of CKD or ESRD patients [10], [30], [31], [36], [37]. Genetic traits and the clustering of shared environmental exposures and health behaviors may contribute to higher FRS and atherosclerotic CVD risk. The application of the FRS may have limitations, since it does not account for other potential predictors for coronary disease. The positive association of relatives of HD patient with MetS and albuminuria strengthens the clinical significance of CVD prediction by FRS.

Furthermore, we have identified increased LDL-C and betel nut chewing as independent determinants for high FRS risk in relatives of HD patients. Dyslipidemia is a well-established risk factor for CVD in the general population, but the causal relationship is much less obvious among CKD and ESRD patients [38], [39]. Betel nut chewing is common in Asian countries, including Taiwan [40]. Previous studies have found that betel nut chewing may activate the sympathetic nervous system, enhance oxidative stress and induce systemic inflammation [41]–[43]. Our findings are consistent with increasing evidence that betel nut chewing is associated with metabolic diseases and CVD [44], [45]. It is essential for physicians to screen a habit of betel nut chewing in their clinical practice. Target treatment for dyslipidemia and development of quit betel nut program might be a key approach of decreasing CVD risk in this population.

We found that familial clustering of metabolic disorders, such as hyperuricemia, hypertension and dyslipidemia, might contribute to MetS, albuminuria, and a higher CVD risk in relatives of HD patients. The causal relationships of these factors are difficult to disentangle. It appears to be an interaction between genetic susceptible traits, environmental factors and behaviors, which are shared to a larger extent than among the controls. Emerging genome-wide association studies may help detecting candidate susceptible genes and facilitate developing new prevention and treatment strategies.

There were several limitations in the current study. First, although our results showed familial aggregation of the risk factors for significant predictors of CVD, additional environmental contributors of CVD, such as low levels of education and lower economic status were not determined in the current study. Family history of a specific disease has been considered to be the consequence of genetic susceptibility, shared environment, and common behaviors [46]. Second, the current study is a cross-sectional design with inherent weaknesses, including the lack of long-term observation for outcomes. Third, the number of study participant is relatively small and this screening study was conducted locally. Fourth, some important factors, such as lifestyles, diet and exercise habits, were lacking. Also, we did not collect the genotypes of HD patients and their relatives. Therefore, we need a more prospective and comprehensive, long-term population-based investigation to clarify CVD risk in family members of ESRD patients.

We conclude that being relatives of HD patients, compared to community controls, was independently associated with MetS, albuminuria and high FRS risk, suggesting family members of ESRD may have a higher CVD risk through the interactions of renal risk factors. Shared environment and familial aggregation may contribute to this excessive CVD risk. Physicians should raise concerns on traditional CVD risk factors as well as on health behaviors and personal habits. We should establish a cessation betel nut chewing program as an interventional action for CVD. Proactive surveillance of these predictors for CVD and preventive strategies should be targeted to this high-risk population.
